# PR interval as a predictor of syncope in tilt-up testing in adolescents and young adults

**DOI:** 10.1186/s43044-021-00149-6

**Published:** 2021-03-20

**Authors:** Juraj Jug, Lada Bradić, Rea Levicki, Martina Lovrić Benčić

**Affiliations:** 1 University of Zagreb School of Medicine, Zagreb, Croatia; 2 University Hospital Zagreb, Zagreb, Croatia; 3 General Hospital Požega, Požega, Croatia

**Keywords:** Syncope, PR interval, Young adults, Autonomic nervous system, Vasovagal, Loss of consciousness

## Abstract

**Background:**

Syncope, as the most frequent consciousness disorder, is very common in young individuals. The aim of this study was to analyze ECG parameters and clinical properties obtained during tilt-up testing in 12 to 30-year-old subjects. We enrolled a total of 142 patients from our outpatient clinic (39 males, 103 females) with a true positive tilt-up test and analyzed ECG records obtained during tilt-testing. Data were stratified according to the age, gender, and type of syncope.

**Results:**

PR interval shortening preceding syncope was found in all syncope types, irrespective of the gender. All types of syncope were more frequent in women (72.5%). Mixed syncope type was found to be the most common (47.18%). Male and female subjects differed in initial heart rate (71.56 vs 76.23/min, *p*=0.05), as well as heart rate dynamics during tilt-up testing. A gender difference was also found in systolic blood pressure (116.92 vs 110.44 mmHg, *p*<0.01), time to syncope onset (20.77 vs. 16.44 min, *p*=0.03), and the total number of syncopal episodes in patient history (2.79 vs. 4.62, *p*<0.05). Subjects with cardioinhibitory syncope had the longest PR interval (average 154.3 ms). PR interval prolongation and loss of variability during tilt-up testing positively correlated with aging (*r*=0.22, *p*<0.05). Nodal rhythm was found in 8 patients.

**Conclusion:**

PR interval shortening on ECG tracings during a tilt-up test can be found in all subtypes of vasovagal syncope, thereby contrasting previous reports that these changes are a hallmark of the cardioinhibitory type of syncope. PR shortening, if observed during ECG monitoring, could be a potential predictor of syncope.

## Background

Neurally mediated or vasovagal syncope is defined as a transient self-limited loss of consciousness due to cerebral hypoperfusion caused by reflex vasodilation, occasionally accompanied by cardioinhibition. Syncope can be preceded by neurovegetative symptoms, and the recovery is usually prompt and complete, regardless of the dramatic onset [1].

Pathophysiologically, neurally mediated syncope results from the stimulation of receptors under control of the autonomic nervous system. Cardiac C fibers react to ventricle volume depletion and increased sympathetic tone in hypovolemia, whereby parasympathetic activation causes reflex bradycardia. This reaction is especially pronounced in adolescents and young adults with autonomic nervous system lability [2, 3]. Prevalence of the neurally mediated syncope in the general population estimates to approximately 22% [4]. Certain studies point to a much higher prevalence of syncope in the young since medical attention in this age is rarely sought [5]. The entire mechanism of syncope pathogenesis has not been elucidated yet.

Neurally mediated syncope is classified according to the new VASIS classification (2000) into mixed (VASIS I), cardioinhibitory with (VASIS IIb) or without asystole (VASIS IIa), and vasodepressor syncope (VASIS III) [6]. By the new VASIS classification, mixed type (VASIS I) became the most common type of syncope [7].

Syncope results from the increased vagal tone, which can also reflect in AV node activation, but the shortening of the PR interval duration is the result of sympathetic activation. Only several papers associate these alterations with the etiopathogenesis of syncope. There are few reports on PR interval duration change on ECG tracing analysis during tilt-up testing, but only in relation to the cardioinhibitory syncope. PR interval reflects conduction through the atria and the AV node [8]. Changes in PR interval duration can result from nervous tone alterations or fibrosis in any of the anatomical structures of the heart, or from pacemaker shift towards the AV node.

The aim of this study was to investigate PR interval changes during tilt-up testing in patients with a positive history for neurally mediated syncope. The focus was put on the PR interval since many patients with frequent syncope showed PR interval shortening in ECG during tilt-up testing.

## Methods

We enrolled a total of 142 consecutive patients (103 females, 39 males), age 12 to 30, admitted to our outpatient clinic for a diagnostic workup of syncope. All these patients had a positive tilt-up test (with true syncope), did not take any medication, and were otherwise healthy. We consider the test as positive if the patient experienced true syncope during the testing and met the criteria for one of the VASIS syncope types. With respect to patient characteristics and to assure a matching number of patients, test subjects were divided into three age subgroups (12–15, 16–18, and 19–30 years). Patients over 30 years were excluded because of a small number of true syncope during tilt-up testing.

ECG tracings were analyzed in D2 lead manually and automatically in all 12 leads during the entire procedure. Manual measurement was performed by ruler, counting PR duration by multiplying PR length in mm with the duration of 1 mm on ECG paper (40 ms). Automatic measurement was performed by *Burdic Atria 6100 electrocardiograph* integrated software. Difference between automatic and manual measurement was insignificant (st. error 15±4 ms). Blood pressure and heart rate measurements were obtained every 5 min for 20 min in the supine, and for 45 more minutes in the upright position. PR interval duration was measured at the beginning of the tilt-up test and on the last visible PR interval in sinus rhythm before syncope occurrence. We intentionally waited until the patient eventual collapse. PR interval was also measured 2 min after the end of the syncope. Each patient with a positive tilt-up test was attributed to a VASIS category, according to syncope characteristics.

### Tilt-up test protocol

All patients were tested from 8:00 am to 12:00 am. At the beginning of the procedure, patients were positioned supine, in ambient temperature of 22–24 °C. Each patient was given 250 mL of 5% glucose intravenously. After 20 min in the supine position, patients were passively tilted to a 75° head-up position without any provocation. The test was aborted either due to syncope provocation or if 45 min passed without syncope. Negative tests were excluded from the study.

### Informed consent

Data was obtained retrospectively from the reports of outpatient tilt-up testing in our clinic. Informed consent was obtained from all participants. Data analysis has been approved by the clinic’s Ethics committee.

### Statistics

All data are expressed as mean ± standard error. Parametric data were compared using an unpaired *t* test. For nonparametric data, we used a Mann-Whitney *U* test. Graphical data, age comparison, and test results were analyzed with multifactorial ANOVA. A value of *p* < 0.05 was considered significant. For a significant change in PR interval duration, we have arbitrarily set the value of 25%. All data were analyzed by Statistica 10.0.

## Results

### Gender differences

**Fig. 1:**
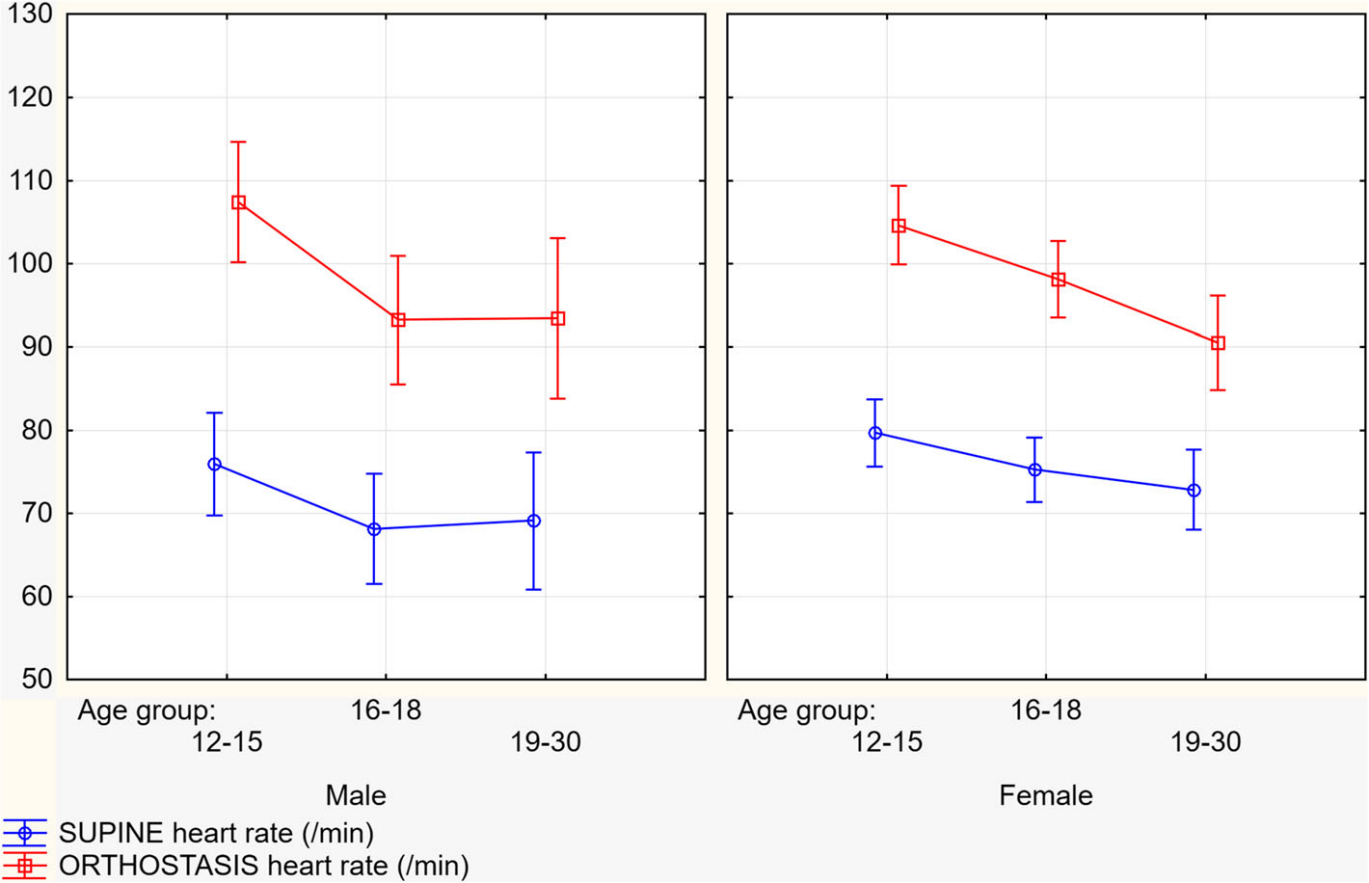
Heart rate dynamics between gender from supine to the upright position during tilt-up test

Mean age of male and female subjects was comparable (16.8 ± 1.2 vs. 17.6 ± 1.1 years, NS), just as their age of the first syncope onset (14.7 vs. 14.8 years, NS). Most of all enrolled patients were female (72.5%), with a history of approximately twice as many syncope episodes (4.62 vs. 2.79, *p*<0.05). In addition, female patients had lower systolic blood pressure at the beginning of the tilt-up test compared to male patients (110.44 vs. 116.92 mmHg, *p*<0.01), earlier the onset of syncope during tilt-up testing (16.44 vs 20.77 min, *p*=0.036), and faster heart rate at the beginning of the test (76.23 vs. 71.56 bpm, *p*=0.05). With table tilting and verticalization more significant increase in heart rate was found in male subjects (27.54 vs. 22.31 bpm, *p*=0.029). However, in the upright the position, we found no difference in heart rate values between genders (99.11 vs. 98.54 bpm, NS) [Fig. 1].

With respect to PR interval shortening greater than 25% from the baseline, female subjects had a PR shortening in VASIS I (12% with <25% vs. 21% with ≥25%, *p*=0.029), but less in VASIS III (16% vs. 11%, *p*=NS). There were no differences between VASIS groups in interval shortening greater than 25% in males.

### VASIS type differences

The most common type of vasovagal syncope was a mixed type (VASIS I, 47.18%), with an age-related increase in the vasodepressor type (VASIS III) from 22.64% in the youngest to 40% of all syncope in the oldest group (Table 1). VASIS II group had significantly longer PR interval when compared to VASIS I (154.3 vs. 142.8 ms, *p*<0.05). The differences between VASIS I and VASIS III groups in PR interval duration existed at the beginning of the testing and remained significant in the upright position until the onset of syncope (Table 2). The average number of syncopal episodes during lifetime was higher in VASIS III group compared to other VASIS groups. PR interval shortening was not found in 6 patients (4.22%).

**Table 1:** Descriptive statistics of VASIS categories according to age

VASIS	Age groups (years)			Total
	12–15 ***n*** = 53	16–18 ***n*** = 54	19–30 ***n*** = 35	12–30 ***n*** = 142
**I**	29 (54.72%)	23 (42.59%)	15 (42.85%)	67 (47.18%)
**IIa**	7 (13.21%)	1 (1.85%)	2 (5.71%)	10 (7.04%)
**IIb**	5 (9.43%)	7 (12.96%)	4 (11.43%)	16 (11.27%)
**III**	12 (22.64%)	23 (42.59%)	14 (40.00%)	49 (34.51%)

**Table 2:** PR interval duration dynamics and average No of syncope in patient history in relation to the VASIS type

	VASIS I	VASIS III	***p***
**Initial PR interval (ms)**	142.8 ± 2.3	150.6 ± 3.2	0.049
**PR before syncope (ms)**	102.9 ± 2.3	114.1 ± 2.8	0.003
**PR interval change ratio (%)**	27.6 ± 1.3	23.8 ± 1.5	0.047
**PR after syncope (ms)**	133.8 ± 2.1	138.7 ± 2.3	0.093
**No of previous syncope**	3.0 ± 0.3	5.7 ± 1.0	0.006

**Fig. 2:**
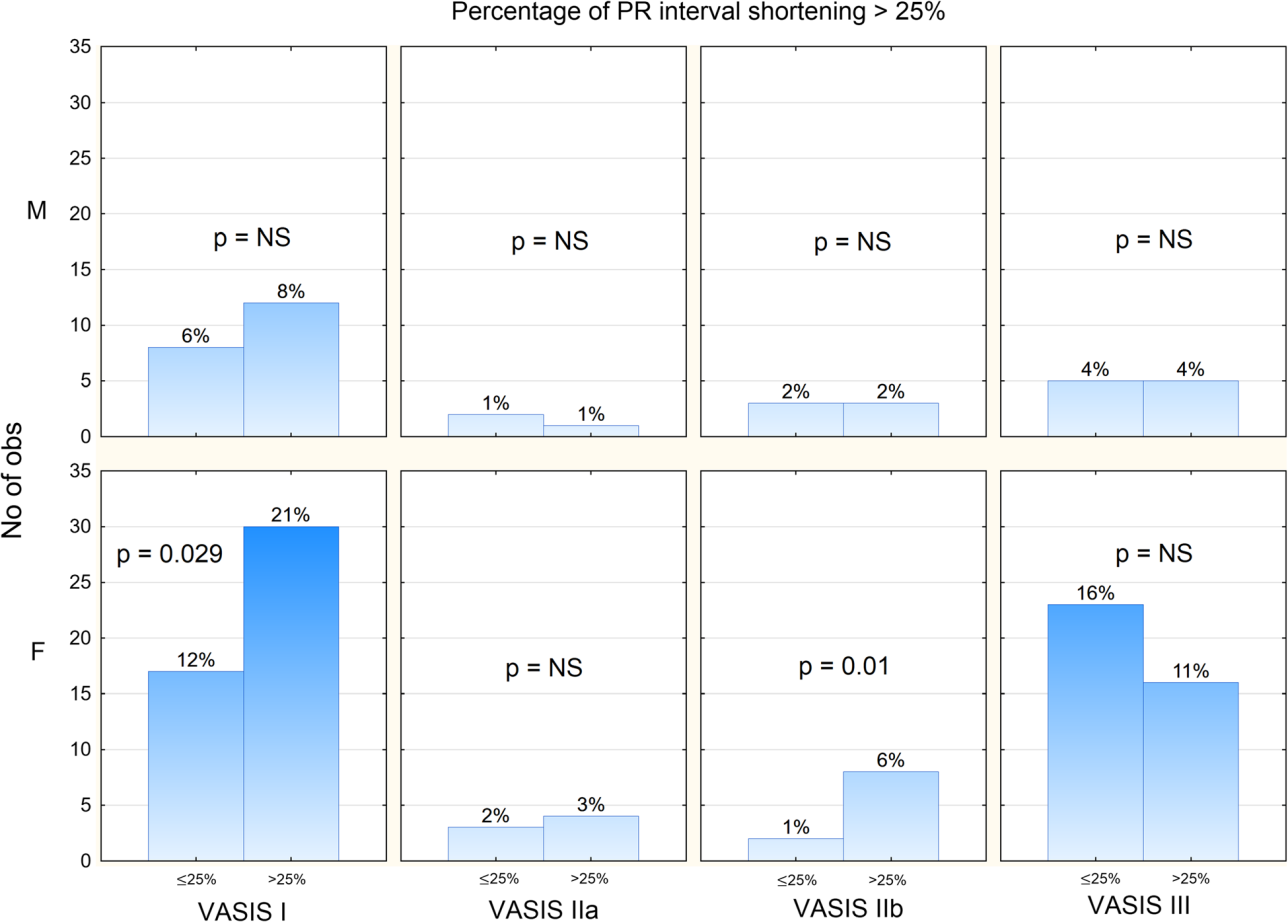
Number and percentage of patients with PR interval change below and above 25% according to gender and VASIS syncope type. (M = male, F = female)

The mean duration of asystole in group IIb was 4.97s (range 3.1 to 7.0s). PR interval change >25% showed no difference between VASIS types in males. In female patients, only VASIS I and IIb showed a difference in the total number of PR interval shortening >25% (Fig. 2).

### Age groups analysis

**Fig. 3:**
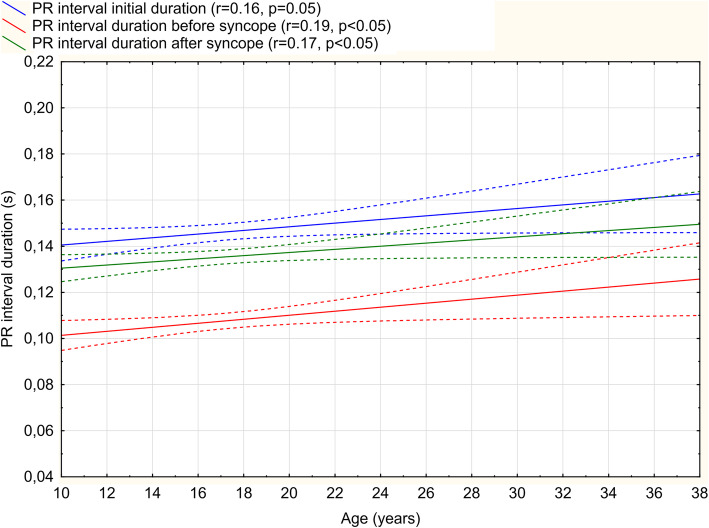
PR interval duration at the beginning of the test, before and after syncope onset according to age

**Fig. 4:**
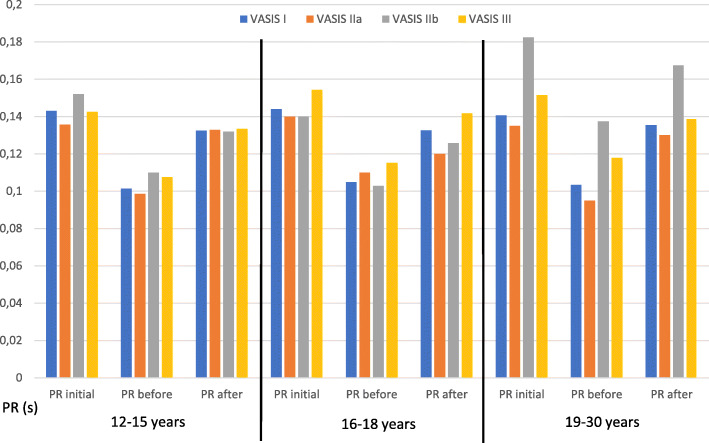
PR interval duration initially, before and after the syncope in different age groups divided by VASIS types

PR interval prolongs with aging (*r*=0.22, *p*<0.05) and the PR change ratio during tilt-up procedure decreases (Table 3 and Fig. 3). Furthermore, the youngest age group had the highest heart rate, in both the supine (78.5 bpm) and the upright position (105.0 bpm) and therefore had the highest heart rate change with a mean of 26.9 bpm from supine to upright. Time to syncope onset was shortest in the oldest age group (16.1 vs. 20.8 min, *p*<0.05). No significant PR interval duration changes initially, before and after the syncope divided by VASIS types between age groups were noticed (Fig. 4).

**Table 3:** Variables according to age groups (BS = before syncope, BP = blood pressure, AS = after syncope)

Variable	Age groups (years)			TOTAL	*p*
	12-15 ***n*** = 53	16-18 ***n*** = 54	19–30 ***n*** = 35	12-30 ***n*** = 142	ANOVA
**Female (%)**	37 (69.81%)	40 (74.07%)	26 (74.28%)	103 (72.53%)	
**PR interval (ms)**	142.8 ± 3.2	147.7 ± 2,7	149.4 ± 3.6	146.3 ± 1.8	0.183
**PR interval BS (ms)**	103.2 ± 2.1	109.1 ± 2.5	112.6 ± 2.5	107.7 ± 1.7	0.046
**PR shortening (%)**	27.6 ± 1.4	25.8 ± 2.3	24.4 ± 1.7	26.1 ± 0.8	0.155
**PR interval AS (ms)**	132.6 ± 1.8	135.4 ± 2.1	140.0 ± 2.2	135.5 ± 2.0	0.185
**QT interval (ms)**	360.6 ± 3.9	362.9 ± 3.5	368.8 ± 3.1	363.5 ± 2.1	0.136
**BMI (kg/m**^**2**^**)**	21.8 ± 1.2	21.2 ± 0.9	22.5 ± 1.1	21.8 ± 0.6	NS
**HR preparation (bpm)**	78.5 ± 1.9	73.4 ± 1.5	71.8 ± 1.9	74.9 ± 1.1	0.023
**HR orthostatic (bpm)**	105 ± 2.2	96.8 ± 1.9	91 ± 2.2	98.7 ± 1.3	0.00003
**HR change (bpm)**	26.9 ± 1.7	23.4 ± 1.6	19.4 ± 2.2	23.7 ± 1.1	0.008
**Time of syncope (min)**	20.8 ± 1.5	20.5 ± 1.5	16.1 ± 1.6	19.6 ± 0.9	0.037

The nodal rhythm was recorded in a total of 11 patients (8 females, 3 males). Although the incidence did not differ between genders (7.7% for both sexes), there was a difference in the percentage of the PR interval change in subjects with nodal rhythm on tilt-up testing in comparison to the sinus rhythm (35.18 vs. 25.32%, *p*<0.001). Its incidence decreased with aging in VASIS I and IIa types (OR = 5.17, *p* = 0.03) in comparison to the VASIS III type, where nodal rhythm was uncommon in all groups.

## Discussion

Thus far, PR changes were described in only a few published papers and in relation strictly to the cardioinhibitory type of syncope (VASIS II). In the pediatric population, tilt-up testing can be very useful in the identification of pseudo-syncope [9]. Other parameters, not routinely measured during tilting, can also aid in defining properties of vasovagal syncope. One of the published papers on PR interval correlation with syncope in young subjects was a paper by Makarov et al. [10]. All the patients with PR interval shortening had a history of syncope, while no changes in QT interval duration was observed. Only one case report was published thus far, with electrophysiologically demonstrated PR interval shortening in a young man with cardioinhibitory syncope, showing PR interval shortening from 142 to 121 ms immediately preceding syncope [11]. Another published paper on the significance of PR interval changes by Mehlsen et al. [12] has shown a reduction of a mean PR interval from 159±28 ms supine to 143±23 ms in the upright position preceding syncope. Our study also demonstrated PR interval shortening in all VASIS types with the biggest differences between VASIS I and III group (Table 2). Seeing the bigger PR shortening, we could predict that syncope would classify as VASIS I in female patients, while males had very similar PR shortening ratios between VASIS groups. PR interval correlation with VASIS type and gender could be interpreted by increased parasympathetic nervous system activity in women (Fig. 2). That was corroborated by several papers of Saleh et al. [13, 14] where intracerebral estrogen injection increased vagal tone in rats, mediated by a high concentration of estrogen receptors in the cardioregulatory neurons of the brain [15]. In addition, estrogen was found to increase muscarinic receptor concentration on the cell membrane [16, 17]. The hearts of female mice were also found to have higher acetylcholine levels than males [18]. This is in the agreement with our results, which demonstrated heart rate change in tilt-testing to be significantly higher in male than in female subjects.

Mechanisms underlying PR shortening in patients with VASIS II type of syncope could be attributed to SA node inhibition by increased vagal tone, as well as a pacemaker shift towards the AV node [11]. Our study suggests that all VASIS types of syncope share a common pathogenesis.

**Fig. 5:**
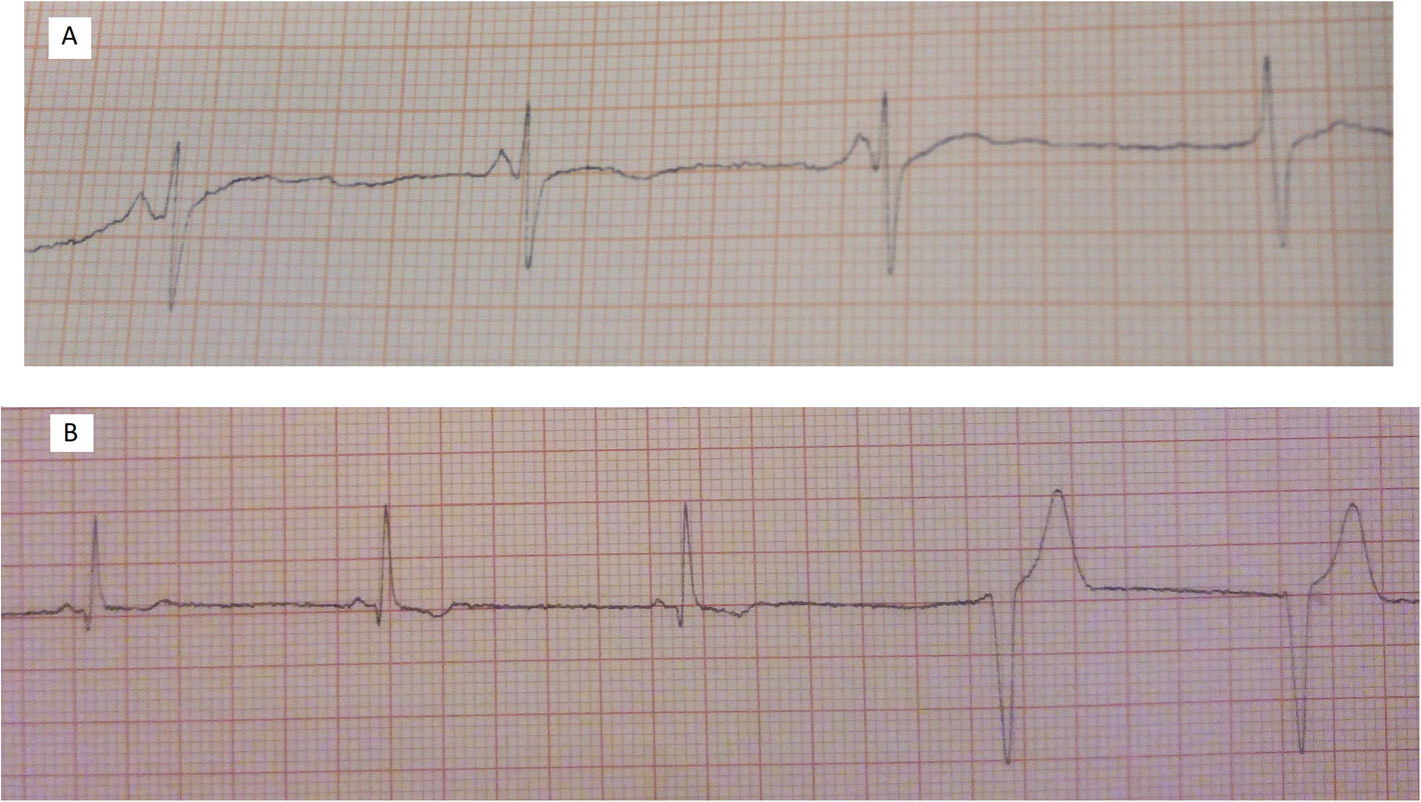
Transition from sinus rhythm to **a** nodal rhythm and **b** idioventricular rhythm

We found rhythm transition from sinus to nodal in 11 patients during the tilt-up test procedure, following the gradual PR shortening. One female patient was found to have an idioventricular rhythm (Fig. 5).

Our results point to a potential common mechanism in VASIS I and II syncope, which essentially differ from the mechanisms in VASIS type III (Table 2). The sensitivity of muscarinic receptors, as well as SA node to the vagal acetylcholine stimulation may be lower in VASIS III syncope, which explains the frequently encountered constant or increased heart rate response in these types of syncope. This could account for a difference in syncope incidence between sexes.

Autonomic cardiovascular regulation decreases with aging, thus potentially accounting for a decrease in the prevalence of VASIS I and II syncope types [3]. In our study, that phenomenon manifested as reduced heart rate dynamics, as well as PR interval prolongation and reduced PR interval shortening rate with aging (Fig. 3). Physiologically, heart rate increases for approximately 30 bpm in young and 10 bpm in older subjects in an upright position [19]. It seems that baroreceptor activity and sensitivity do not differ between pediatric and young adult patients [20]. However, baroreceptor sensitivity decrease can be noticed in adulthood, with increasing age [21]. Changes in PR interval and heart rate variability in younger population can be attributed to incomplete autonomic nervous system maturation. The physiology of the autonomic nervous system maturation has not been entirely elucidated yet and potentially relates to both mechanical and neural alterations with aging [22].

Even though we suggest that PR interval shortening could be an important predictor of syncope (4.22% of patients (6/142) did not show PR shortening in ECG), a tilt-up test should be performed completely because of unknown sensibility and specificity for this change. A control group could not be assessed because if syncope is absent the PR measurement (done at the moment before syncope) is impossible.

## Conclusion

PR interval shortening on ECG tracings during a tilt-up test can be found in all subtypes of vasovagal syncope, thereby contrasting previous reports that these changes are a hallmark of the cardioinhibitory type of syncope. Patients with the VASIS III group had the highest number of syncopal episodes and the longest PR interval duration, and their PR interval shortening was the smallest among all VASIS types. PR interval shortening differed mostly between VASIS I and VASIS III syncope. We suggest that PR shortening, if observed during ECG monitoring, could be a possible predictor of syncope. Further investigation is needed to confirm the significance of these findings.

### Study limitations

PR interval changes were measured automatically and checked manually on ECG tracings of tilt-table tests performed on outpatients younger than 30 years in our clinic because of a very small number of positive results in older than 30 years. Specificity and the sensitivity of PR interval duration changes in syncope episodes are unknown because PR interval shortening measurements in the control group are impossible.

## Data Availability

The datasets used and/or analyzed during the current study are available from the corresponding author on reasonable request.

## Abbreviations

ECG: Electrocardiogram
AV: Atrioventricular
SA: Sinoatrial
BMI: Body mass index
HR: Heart rate
BS: Before syncope
BP: Blood pressure
BPM: Beats per minute
